# Bis(1,3-dibutylthiourea)dicyanido­mercury(II)

**DOI:** 10.1107/S1600536809035594

**Published:** 2009-09-09

**Authors:** Saeed Ahmad, Haseeba Sadaf, Mehmet Akkurt, Shahzad Sharif, Islam Ullah Khan

**Affiliations:** a Department of Chemistry, University of Engineering and Technology, Lahore 54890, Pakistan; b Department of Physics, Faculty of Arts and Sciences, Erciyes University, 38039 Kayseri, Turkey; c Materials Chemistry Laboratory, Department of Chemistry, Government College University, Lahore 54000, Pakistan

## Abstract

In the title compound, [Hg(CN)_2_(C_9_H_20_N_2_S)_2_], the Hg atom lies on a twofold rotation axis. There is only half a mol­ecule in the asymmetric unit. The Hg atom has a distorted tetra­hedral coordination involving the S atoms of two 1-butyl-3-propyl­thio­urea groups and the C atoms of the two CN^−^ anions. In the crystal packing, adjacent mol­ecules are connected by inter­molecular N—H⋯N and N—H⋯S hydrogen bonds, forming infinite chains in three dimensions.

## Related literature

For the coordination chemistry of thio­urea-type ligands, see: Nadeem *et al.* (2009 ▶, 2008 ▶); Zoufalá *et al.* (2007 ▶); Khan *et al.* (2007 ▶); Hanif *et al.* (2007 ▶); Fuks *et al.* (2005 ▶); Moro *et al.* (2009 ▶); Matesanz & Souza (2007 ▶). For crystallographic reports about mercury(II) complexes containing thio­amides, see: Popovic *et al.* (2000 ▶, 2002 ▶); Pavlović *et al.* (2000 ▶); Jiang *et al.* (2001 ▶); Wu *et al.* (2004 ▶). For the spectroscopy and structural chemistry of cyanide complexes of silver(I) and gold(I) with thio­nes, see: Hanif *et al.* (2007 ▶); Wu *et al.* (2004 ▶); Ahmad, Isab & Ashraf (2002 ▶); Ahmad, Isab & Perzanowski (2002 ▶); Ashraf *et al.* (2002 ▶); Ahmad & Isab (2001 ▶); Ahmad (2004 ▶).
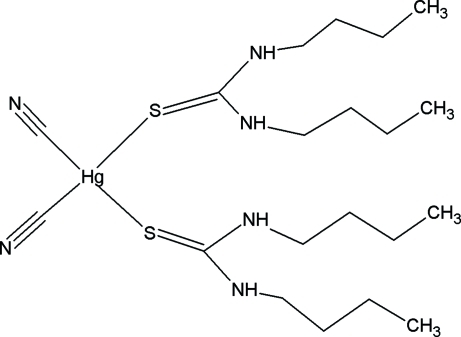


## Experimental

### 

#### Crystal data

[Hg(CN)_2_(C_9_H_20_N_2_S)_2_]
*M* *_r_* = 629.31
Monoclinic, 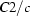

*a* = 17.4692 (3) Å
*b* = 9.5928 (2) Å
*c* = 17.4699 (4) Å
β = 111.540 (1)°
*V* = 2723.12 (10) Å^3^
*Z* = 4
Mo *K*α radiation
μ = 5.82 mm^−1^
*T* = 296 K
0.14 × 0.15 × 0.17 mm

#### Data collection

Bruker Kappa APEXII CCD area-detector diffractometer
Absorption correction: none
15120 measured reflections
3372 independent reflections
2918 reflections with *I* > 2σ(*I*)
*R*_int_ = 0.032

#### Refinement

*R*[*F* ^2^ > 2σ(*F* ^2^)] = 0.022
*wR*(*F* ^2^) = 0.044
*S* = 1.02
3372 reflections
134 parameters
H-atom parameters constrained
Δρ_max_ = 0.40 e Å^−3^
Δρ_min_ = −0.72 e Å^−3^

### 

Data collection: *APEX2* (Bruker, 2007 ▶); cell refinement: *SAINT* (Bruker, 2007 ▶); data reduction: *SAINT*; program(s) used to solve structure: *SHELXS97* (Sheldrick, 2008 ▶); program(s) used to refine structure: *SHELXL97* (Sheldrick, 2008 ▶); molecular graphics: *ORTEP-3 for Windows* (Farrugia, 1997 ▶); software used to prepare material for publication: *WinGX* (Farrugia, 1999 ▶) and *PLATON* (Spek, 2009 ▶).

## Supplementary Material

Crystal structure: contains datablocks global, I. DOI: 10.1107/S1600536809035594/bt5052sup1.cif

Structure factors: contains datablocks I. DOI: 10.1107/S1600536809035594/bt5052Isup2.hkl

Additional supplementary materials:  crystallographic information; 3D view; checkCIF report

## Figures and Tables

**Table 1:** Hydrogen-bond geometry (Å, °)

*D*—H⋯*A*	*D*—H	H⋯*A*	*D*⋯*A*	*D*—H⋯*A*
N2—H2⋯S1^i^	0.86	2.68	3.479 (2)	155
N3—H3⋯N1^ii^	0.86	2.20	2.991 (3)	153
C7—H7*B*⋯S1	0.97	2.67	3.070 (3)	105

Symmetry codes: (i) 
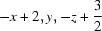
; (ii) 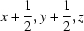.

**Fig. 1.**
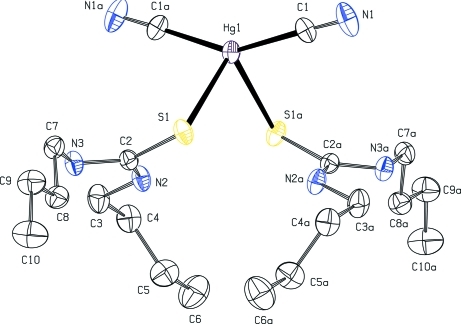
The title molecule with the atom numbering scheme. Displacement ellipsoids for non-H atoms are drawn at the 30% probability level.

**Fig. 2.**
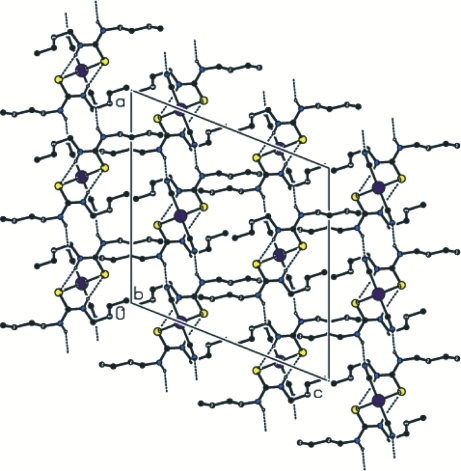
The packing and hydrogen bonding of (I) viewed down b axis. Hydrogen atoms not involved in the showed interactions have been omitted for clarity.

## supplementary crystallographic information

### Comment

The coordination chemistry of thiourea type ligands has been the subject of several recent studies because of the relevance of their binding sites to those in living systems (Nadeem *et al.*, 2009; Nadeem *et al.*, 2008; Zoufalá *et al.*, 2007; Khan *et al.*, 2007; Hanif *et al.*, 2007; Fuks *et al.*, 2005; Moro *et al.*, 2009; Matesanz & Souza, 2007). Crystallographic reports about mercury (II) complexes containing thioamides establish that these ligands are coordinated *via* the sulfur atom(Popovic *et al.*, 2000, 2002; Pavlović, Popović, Soldin *et al.*, 2000; Jiang *et al.*, 2001; Wu *et al.*, 2004). We have been involved in investigating the spectral and structural chemistry of cyanido complexes of silver (I) and gold (I) with thiones with emphasis onligand scrambling reactions (Hanif *et al.*, 2007; Wu *et al.*, 2004; Ahmad, Isab & Ashraf, 2002; Ahmad, Isab & Perzanowski, 2002; Ashraf *et al.*, 2002; Ahmad & Isab, 2001; Ahmad, 2004). As a part ofextension of our work towards complexation of Hg (CN)_2_ with thiones, we report here the crystal structures of [(*N*,*N*^/^-dibutylthiourea)_2_Hg(CN)_2_], (I).

In the title compound (I), (Fig. 1), the Hg anion lies on a twofold rotation axes paralel to the *b* axis in space group *C*2/*c* and one half of the molecule to the other half are connected by this symmetry operation. The Hg atom has a distorted tetrahedral coordination by the S atoms of two *N*,*N*^/^-dibutylthiourea groups and the C atoms of the two CN groups. The bond distances Hg—S and Hg—C are 2.7424 (7) Å and 2.072 (3) Å, and the bond angles C—Hg—C, S—Hg—S and C—Hg—C are 150.51 (11)°, 95.55 (2)° and 100.45 (8)°. All bond lengths and bond angles in (I) are in the range of expected values.

In the crystal packing, the adjacent molecules are connected by intermolecular N—H···N and N—H···S hydrogen bonds (Table 1). In Fig. 2, the packing and hydrogen bonding of (I) are shown viewed down *b* axis.

### Experimental

For the preparation of the title complex, Hg (CN)_2_ was prepared first by the reaction of 1 mmol HgCl_2_ in methanol with 2 mmole of KCN in water. Then 0.253 g (1 mmole) Hg (CN)_2_ dissolved in15 ml methanol was mixed with 2 equivalents of *N*,*N*^/^-dibutylthiourea in 15 methanol. After stirring for 15 minutes, the resulting mixture was filtered and filtrate was kept at room temperature. After 24 h white crystals were obtained.

### Refinement

H atoms were located geometrically and treated as riding with C—H = 0.97 Å (methylene), C—H = 0.96 Å (methyl) and N—H = 0.86 Å with *U*_iso_(H) = 1.2 or 1.5*U*_eq_(C, N).

### Crystal data

**Table:**

[Hg(CN)_2_(C_9_H_20_N_2_S)_2_]	*F*(000) = 1256
*M**_r_* = 629.31	*D*_x_ = 1.535 Mg m^−^^3^
Monoclinic, *C*2/*c*	Mo *K*α radiation, λ = 0.71073 Å
Hall symbol: -C 2yc	Cell parameters from 6609 reflections
*a* = 17.4692 (3) Å	θ = 2.5–27.2°
*b* = 9.5928 (2) Å	µ = 5.82 mm^−^^1^
*c* = 17.4699 (4) Å	*T* = 296 K
β = 111.540 (1)°	Irregular, white
*V* = 2723.12 (10) Å^3^	0.17 × 0.15 × 0.14 mm
*Z* = 4	

### Data collection

**Table:**

Bruker Kappa APEXII CCD area-detector diffractometer	2918 reflections with *I* > 2σ(*I*)
Radiation source: sealed tube	*R*_int_ = 0.032
graphite	θ_max_ = 28.3°, θ_min_ = 2.5°
φ and ω scans	*h* = −23→23
15120 measured reflections	*k* = −12→12
3372 independent reflections	*l* = −20→23

### Refinement

**Table:**

Refinement on *F*^2^	Primary atom site location: structure-invariant direct methods
Least-squares matrix: full	Secondary atom site location: difference Fourier map
*R*[*F*^2^ > 2σ(*F*^2^)] = 0.022	Hydrogen site location: inferred from neighbouring sites
*wR*(*F*^2^) = 0.044	H-atom parameters constrained
*S* = 1.02	*w* = 1/[σ^2^(*F*_o_^2^) + (0.0211*P*)^2^], where *P* = (*F*_o_^2^ + 2*F*_c_^2^)/3
3372 reflections	(Δ/σ)_max_ = 0.001
134 parameters	Δρ_max_ = 0.40 e Å^−^^3^
0 restraints	Δρ_min_ = −0.72 e Å^−^^3^

### Special details

**Table:**

Geometry. Bond distances, angles *etc*. have been calculated using the rounded fractional coordinates. All su's are estimated from the variances of the (full) variance-covariance matrix. The cell e.s.d.'s are taken into account in the estimation of distances, angles and torsion angles
Refinement. Refinement on *F*^2^ for ALL reflections except those flagged by the user for potential systematic errors. Weighted *R*-factors *wR* and all goodnesses of fit *S* are based on *F*^2^, conventional *R*-factors *R* are based on *F*, with *F* set to zero for negative *F*^2^. The observed criterion of *F*^2^ > σ(*F*^2^) is used only for calculating -*R*-factor-obs *etc*. and is not relevant to the choice of reflections for refinement. *R*-factors based on *F*^2^ are statistically about twice as large as those based on *F*, and *R*-factors based on ALL data will be even larger.

### Fractional atomic coordinates and isotropic or equivalent isotropic displacement parameters (Å^2^)

**Table:**

	*x*	*y*	*z*	*U*_iso_*/*U*_eq_	
Hg1	1.00000	0.20478 (1)	0.75000	0.0405 (1)	
S1	1.08516 (3)	0.39691 (7)	0.86634 (4)	0.0414 (2)	
N1	0.86048 (15)	0.1224 (3)	0.81955 (17)	0.0718 (10)	
N2	1.11488 (12)	0.5094 (2)	0.74219 (12)	0.0422 (7)	
N3	1.22801 (11)	0.4758 (2)	0.85840 (12)	0.0411 (7)	
C1	0.91098 (16)	0.1498 (3)	0.79668 (17)	0.0475 (9)	
C2	1.14750 (13)	0.4655 (2)	0.81878 (15)	0.0341 (7)	
C3	1.16093 (17)	0.5591 (3)	0.69240 (17)	0.0548 (10)	
C4	1.10347 (17)	0.6051 (3)	0.60900 (17)	0.0534 (10)	
C5	1.0548 (2)	0.7334 (4)	0.6094 (2)	0.0633 (12)	
C6	0.9979 (3)	0.7767 (4)	0.5243 (3)	0.0875 (17)	
C7	1.27223 (15)	0.4387 (3)	0.94375 (16)	0.0441 (8)	
C8	1.27906 (17)	0.5571 (3)	1.00242 (16)	0.0473 (9)	
C9	1.3311 (2)	0.5232 (3)	1.09034 (17)	0.0586 (11)	
C10	1.3434 (3)	0.6466 (4)	1.1477 (2)	0.0795 (14)	
H2	1.06210	0.50880	0.71950	0.0510*	
H3	1.25650	0.50710	0.83120	0.0490*	
H3A	1.19600	0.48500	0.68630	0.0660*	
H3B	1.19580	0.63660	0.72020	0.0660*	
H4A	1.13530	0.62230	0.57470	0.0640*	
H4B	1.06550	0.52970	0.58410	0.0640*	
H5A	1.09240	0.80930	0.63420	0.0760*	
H5B	1.02240	0.71650	0.64330	0.0760*	
H6A	1.02880	0.78550	0.48910	0.1310*	
H6B	0.97300	0.86460	0.52750	0.1310*	
H6C	0.95580	0.70750	0.50240	0.1310*	
H7A	1.32710	0.40780	0.95010	0.0530*	
H7B	1.24430	0.36120	0.95810	0.0530*	
H8A	1.22430	0.58250	0.99950	0.0570*	
H8B	1.30250	0.63730	0.98510	0.0570*	
H9A	1.38450	0.49040	1.09270	0.0700*	
H9B	1.30520	0.44820	1.10920	0.0700*	
H10A	1.36760	0.72220	1.12850	0.1190*	
H10B	1.37910	0.62040	1.20210	0.1190*	
H10C	1.29110	0.67540	1.14870	0.1190*	

### Atomic displacement parameters (Å^2^)

**Table:**

	*U*^11^	*U*^22^	*U*^33^	*U*^12^	*U*^13^	*U*^23^
Hg1	0.0323 (1)	0.0459 (1)	0.0478 (1)	0.0000	0.0199 (1)	0.0000
S1	0.0359 (3)	0.0566 (4)	0.0340 (3)	−0.0115 (3)	0.0154 (3)	−0.0005 (3)
N1	0.0516 (14)	0.104 (2)	0.0668 (17)	−0.0297 (15)	0.0300 (13)	−0.0046 (17)
N2	0.0326 (10)	0.0594 (14)	0.0352 (12)	−0.0068 (9)	0.0133 (9)	0.0057 (11)
N3	0.0313 (10)	0.0545 (13)	0.0383 (12)	−0.0043 (9)	0.0136 (9)	0.0051 (10)
C1	0.0385 (13)	0.0553 (16)	0.0463 (16)	−0.0125 (12)	0.0126 (12)	−0.0037 (14)
C2	0.0330 (11)	0.0369 (13)	0.0340 (14)	−0.0030 (9)	0.0142 (10)	−0.0015 (11)
C3	0.0473 (15)	0.076 (2)	0.0491 (17)	−0.0018 (14)	0.0270 (14)	0.0144 (15)
C4	0.0563 (16)	0.069 (2)	0.0396 (16)	−0.0019 (14)	0.0232 (13)	0.0039 (15)
C5	0.076 (2)	0.066 (2)	0.052 (2)	0.0065 (17)	0.0285 (17)	0.0103 (16)
C6	0.084 (3)	0.101 (3)	0.074 (3)	0.026 (2)	0.025 (2)	0.020 (2)
C7	0.0353 (12)	0.0476 (15)	0.0446 (16)	0.0040 (11)	0.0089 (11)	0.0054 (13)
C8	0.0477 (14)	0.0466 (15)	0.0427 (16)	0.0038 (12)	0.0110 (12)	0.0064 (13)
C9	0.0665 (19)	0.0565 (19)	0.0433 (17)	0.0044 (15)	0.0090 (15)	0.0050 (15)
C10	0.107 (3)	0.066 (2)	0.051 (2)	−0.005 (2)	0.012 (2)	−0.0008 (19)

### Geometric parameters (Å, °)

**Table:**

Hg1—S1	2.7424 (7)	C3—H3A	0.9700
Hg1—C1	2.072 (3)	C3—H3B	0.9700
Hg1—S1^i^	2.7424 (7)	C4—H4A	0.9700
Hg1—C1^i^	2.072 (3)	C4—H4B	0.9700
S1—C2	1.724 (2)	C5—H5A	0.9700
N1—C1	1.125 (4)	C5—H5B	0.9700
N2—C2	1.316 (3)	C6—H6A	0.9600
N2—C3	1.465 (4)	C6—H6B	0.9600
N3—C2	1.324 (3)	C6—H6C	0.9600
N3—C7	1.449 (3)	C7—H7A	0.9700
N2—H2	0.8600	C7—H7B	0.9700
N3—H3	0.8600	C8—H8A	0.9700
C3—C4	1.500 (4)	C8—H8B	0.9700
C4—C5	1.497 (5)	C9—H9A	0.9700
C5—C6	1.512 (6)	C9—H9B	0.9700
C7—C8	1.505 (4)	C10—H10A	0.9600
C8—C9	1.505 (4)	C10—H10B	0.9600
C9—C10	1.514 (5)	C10—H10C	0.9600
			
S1···C1	3.693 (3)	H3A···N3	2.8500
S1···C8	3.684 (3)	H3A···H3	2.3700
S1···N2^i^	3.479 (2)	H3A···H10C^vi^	2.5200
S1···H7B	2.6700	H3B···N3	2.7400
S1···H2^i^	2.6800	H3B···H3	2.2200
S1···H6A^ii^	3.1900	H3B···H5A	2.5000
N1···N3^iii^	2.991 (3)	H3B···N1^iv^	2.7600
N1···C3^iii^	3.431 (4)	H4A···H6A	2.4700
N2···S1^i^	3.479 (2)	H4B···H2	2.4000
N3···N1^iv^	2.991 (3)	H4B···H6C	2.5700
N1···H3B^iii^	2.7600	H5A···H3B	2.5000
N1···H3^iii^	2.2000	H5A···H7A^vii^	2.5600
N2···H5B	2.7400	H5B···N2	2.7400
N3···H3A	2.8500	H5B···H2	2.3500
N3···H3B	2.7400	H6A···H4A	2.4700
C1···S1	3.693 (3)	H6A···S1^vi^	3.1900
C1···C2^i^	3.574 (4)	H6C···H4B	2.5700
C3···N1^iv^	3.431 (4)	H7A···H9A	2.4500
C8···S1	3.684 (3)	H7A···H5A^viii^	2.5600
C1···H10B^v^	3.0100	H7B···S1	2.6700
C3···H3	2.4400	H7B···H9B	2.6000
C5···H2	2.8600	H7B···H7B^ix^	2.5500
H2···C5	2.8600	H8A···H10C	2.5900
H2···H4B	2.4000	H8B···H10A	2.4800
H2···H5B	2.3500	H9A···H7A	2.4500
H2···S1^i^	2.6800	H9B···H7B	2.6000
H3···C3	2.4400	H10A···H8B	2.4800
H3···H3A	2.3700	H10B···C1^x^	3.0100
H3···H3B	2.2200	H10C···H8A	2.5900
H3···N1^iv^	2.2000	H10C···H3A^ii^	2.5200
			
S1—Hg1—C1	99.25 (8)	C4—C5—H5A	109.00
S1—Hg1—S1^i^	95.55 (2)	C4—C5—H5B	109.00
S1—Hg1—C1^i^	100.45 (8)	C6—C5—H5A	109.00
S1^i^—Hg1—C1	100.45 (8)	C6—C5—H5B	109.00
C1—Hg1—C1^i^	150.51 (11)	H5A—C5—H5B	108.00
S1^i^—Hg1—C1^i^	99.25 (8)	C5—C6—H6A	109.00
Hg1—S1—C2	99.56 (8)	C5—C6—H6B	109.00
C2—N2—C3	125.5 (2)	C5—C6—H6C	109.00
C2—N3—C7	125.6 (2)	H6A—C6—H6B	109.00
C2—N2—H2	117.00	H6A—C6—H6C	110.00
C3—N2—H2	117.00	H6B—C6—H6C	109.00
C2—N3—H3	117.00	N3—C7—H7A	109.00
C7—N3—H3	117.00	N3—C7—H7B	109.00
Hg1—C1—N1	177.4 (3)	C8—C7—H7A	109.00
S1—C2—N2	119.84 (19)	C8—C7—H7B	109.00
S1—C2—N3	120.95 (18)	H7A—C7—H7B	108.00
N2—C2—N3	119.2 (2)	C7—C8—H8A	109.00
N2—C3—C4	110.8 (2)	C7—C8—H8B	109.00
C3—C4—C5	114.6 (2)	C9—C8—H8A	109.00
C4—C5—C6	113.0 (3)	C9—C8—H8B	109.00
N3—C7—C8	113.2 (2)	H8A—C8—H8B	108.00
C7—C8—C9	113.5 (2)	C8—C9—H9A	109.00
C8—C9—C10	113.1 (3)	C8—C9—H9B	109.00
N2—C3—H3A	109.00	C10—C9—H9A	109.00
N2—C3—H3B	109.00	C10—C9—H9B	109.00
C4—C3—H3A	109.00	H9A—C9—H9B	108.00
C4—C3—H3B	109.00	C9—C10—H10A	109.00
H3A—C3—H3B	108.00	C9—C10—H10B	109.00
C3—C4—H4A	109.00	C9—C10—H10C	109.00
C3—C4—H4B	109.00	H10A—C10—H10B	110.00
C5—C4—H4A	109.00	H10A—C10—H10C	109.00
C5—C4—H4B	109.00	H10B—C10—H10C	110.00
H4A—C4—H4B	108.00		
			
C1—Hg1—S1—C2	−169.87 (11)	C7—N3—C2—S1	−2.8 (3)
S1^i^—Hg1—S1—C2	−68.30 (8)	C7—N3—C2—N2	176.7 (2)
C1^i^—Hg1—S1—C2	32.19 (11)	C2—N3—C7—C8	−88.9 (3)
Hg1—S1—C2—N2	51.24 (18)	N2—C3—C4—C5	67.9 (3)
Hg1—S1—C2—N3	−129.28 (17)	C3—C4—C5—C6	179.8 (3)
C3—N2—C2—S1	−174.74 (19)	N3—C7—C8—C9	−175.0 (2)
C3—N2—C2—N3	5.8 (3)	C7—C8—C9—C10	175.2 (3)
C2—N2—C3—C4	−178.1 (2)		

Symmetry codes: (i) −*x*+2, *y*, −*z*+3/2; (ii) *x*, −*y*+1, *z*+1/2; (iii) *x*−1/2, *y*−1/2, *z*; (iv) *x*+1/2, *y*+1/2, *z*; (v) *x*−1/2, −*y*+1/2, *z*−1/2; (vi) *x*, −*y*+1, *z*−1/2; (vii) −*x*+5/2, *y*+1/2, −*z*+3/2; (viii) −*x*+5/2, *y*−1/2, −*z*+3/2; (ix) −*x*+5/2, −*y*+1/2, −*z*+2; (x) *x*+1/2, −*y*+1/2, *z*+1/2.

### Hydrogen-bond geometry (Å, °)

**Table:**

*D*—H···*A*	*D*—H	H···*A*	*D*···*A*	*D*—H···*A*
N2—H2···S1^i^	0.86	2.68	3.479 (2)	155
N3—H3···N1^iv^	0.86	2.20	2.991 (3)	153
C7—H7B···S1	0.97	2.67	3.070 (3)	105

Symmetry codes: (i) −*x*+2, *y*, −*z*+3/2; (iv) *x*+1/2, *y*+1/2, *z*.
